# Chelating, Reducing, and Adsorbing Agents in Geopolymers for Heavy Metals Stabilization from Galvanic Sludge

**DOI:** 10.3390/polym18010028

**Published:** 2025-12-22

**Authors:** Francesco Genua, Mattia Giovini, Cristina Leonelli, Isabella Lancellotti

**Affiliations:** Department of Engineering “Enzo Ferrari”, University of Modena and Reggio Emilia, Via Pietro Vivarelli 10, 41125 Modena, Italy; mattia.giovini@unimore.it (M.G.); cristina.leonelli@unimore.it (C.L.)

**Keywords:** geopolymer, heavy metal immobilization, stabilization/solidification (S/S), galvanic sludge waste, chelating agents, reducing agents

## Abstract

Hazardous galvanic sludge waste (GSW) from the electroplating industry, produced at 100,000–150,000 tonnes/year in the EU and containing high concentrations of Cr and Ni was successfully treated using metakaolin-based geopolymers via Stabilization/Solidification (S/S). The experimental design incorporated chelating (sodium diethyl dithio carbamate, C_5_H_10_NS_2_Na, DTC), reducing (sodium sulfide, Na_2_S), and adsorbing (hydroxyapatite, Ca_5_(PO_4_)_3_(OH), Hap) agents separately to improve heavy metal immobilization. The results demonstrated that Na_2_S drastically decreased Cr release by −98.7% by reducing mobile Cr(VI) to insoluble Cr(III). DTC reduced Ni leaching by −93.4%, forming sparingly soluble Ni(II)(DTC)_2_ complexes that precipitated within the matrix. Hap enhanced Ni retention by 55.5% via cation exchange but was ineffective for Cr due to electrostatic repulsion with the anion Cr(VI)O_4_^2−^ at the geopolymer’s high pH. This work is the first to apply geopolymerization coupled with these chemical agents for S/S of as-received galvanic waste, offering a highly efficient, low-carbon strategy to manage this hazardous industrial residue.

## 1. Introduction

Metal plating processes generate large quantities of hazardous waste. These operations involve wastewater treatment, leading to the production of Galvanic Sludge Wastes (GSW), produced at 100,000–150,000 tonnes/year across EU countries [1,2]. The disposal method in specialized landfills leads to economic burdens and contributes to the accumulation of hazardous materials in the environment [2]. GSW is primarily composed of metal hydroxides and sulfates, and contains high concentrations of heavy metals, particularly chromium (Cr) and nickel (Ni). A critical environmental concern is the presence of trivalent chromium (Cr(III)), which can oxidize to the highly water-soluble and carcinogenic hexavalent chromium (Cr(VI)) under specific conditions [1,3,4].

Treatment and recycling options have been investigated to limit GSW environmental impact. Hydrometallurgical techniques focus on recovering metals such as nickel, copper, and zinc [5]. Solidification/stabilization (S/S) remains a widely employed approach for the long-term management of hazardous residues. The aim of S/S is to improve the physical properties of the waste, reducing leachability and limiting heavy metal solubility within a robust solid matrix [6].

Various S/S techniques have been investigated, such as incorporation into clay matrices for ceramic brick production [1], stabilization with asphalt emulsions [7], and vitrification [8]. These methods show variable effectiveness, but they require high-temperature processes, typically around 1400 °C for the clinker production [7], 950 °C for ceramics [1] and even higher for glass formation [8], which result in significant energy consumption and associated CO_2_ emissions.

Geopolymers represent a more sustainable alternative to conventional binders [9]. These aluminosilicate-based materials, obtained through alkali activation of natural aluminosilicate precursors such as metakaolin [10] or industrial by-products such as fly ash and blast furnace slag [11,12], can be synthesized at room temperature or below 80 °C [13,14]. Geopolymerization involves the alkali activation of aluminosilicate precursors, which undergo dissolution, polycondensation and solidification. Metakaolin, specifically, is a highly reactive aluminosilicate precursor due to its structural disorder and high content of amorphous phases resulting from the dehydroxylation of kaolinite. This amorphous nature provides a high density of readily soluble Si and Al species, which promotes rapid and uniform geopolymer gel formation, a crucial characteristic for effectively encapsulating metal-rich wastes like galvanic sludge and ensuring a homogeneous matrix [15].

Dissolution: In alkaline media, SiO_2_ and Al_2_O_3_ dissolve into Si(OH)_4_ and Al(OH)_4_^−^, as OH^−^ breaks Si–O–Si and Al–O–Si bonds in the precursors [16,17]. Solid-state ^27^Al MAS NMR revealed that alkali activation of metakaolin promotes the conversion of octahedral [Al(VI)] and pentahedral [Al(V)] species into tetrahedral [Al(IV)] units. While pristine metakaolin contains comparable amounts of Al(IV), Al(V), and Al(VI), reacted products are dominated by Al(IV), charge-balanced by alkali cations [10].
Polycondensation: Reactive monomers condense into ≡Si–O–Al≡ and Si–O–Si linkages with water release:
  Si(OH)_4_ + Al(OH)_4_^−^ →  ≡T–O–T≡ + H_2_O (T=Si or Al^−^(IV))

This gel network develops into an amorphous 3D matrix, governed by the Si/Al ratio and curing conditions [11].

Solidification: Final consolidation occurs as water is partially expelled, yielding a dense matrix [10].

Life cycle assessments have reported reductions in CO_2_-equivalent emissions of up to 80% compared with ordinary Portland cement containing clinker [9]. In addition, their three-dimensional aluminosilicate network provides reactive binding sites for heavy metals, improving immobilization efficiency [18].

The immobilization of heavy metals within geopolymer matrices occurs through a variety of chemical and physical interactions, which are collectively classified under the Solidification/Stabilization processes [19]. The unique three-dimensional framework of geopolymers supports both stabilization and adsorption processes. The stabilization mechanisms inherent to the geopolymer matrix are:

1. Physical Encapsulation (Solidification):
  This involves the physical entrapment of heavy metal ions within the dense, compact, cross-linked, three-dimensional structure (framework) of the amorphous geopolymer gel [17]. This structural barrier restricts the movement and leaching potential of the contaminants. Due to the dense and compact nature of the geopolymers, they create a barrier that restricts the movement of contaminants [17,18].
2. Precipitation:
  Geopolymerization occurs in a highly alkaline environment (typically pH > 11), which promotes the formation of insoluble metal compounds, such as hydroxides, carbonates, or phosphates. This significantly reduces the solubility and mobility of the heavy metals within the matrix. Pb and Cr have been observed to precipitate as metal hydroxides (Pb(OH)_2_ and Cr(OH)_3_) in the alkaline environment [17,18,20].
3. Ion Exchange:
  The aluminosilicate framework contains tetrahedrally coordinated aluminum ions (Al^3+^), which create a negative charge on the [Al(OH)_4_]^−^ units. To maintain charge neutrality, positively charged heavy metal cations (such as Pb^2+^, Cd^2+^ and Cu^2+^) can be incorporated into the structure by replacing the charge-balancing alkali cations (Na^+^ or K^+^) [18,20,21].
4. Isomorphic Substitution:
  This mechanism involves metal cations (such as Pb^2+^, Cu^2+^ and Zn^2+^) replacing the tetrahedral Al^3+^ ions within the aluminosilicate framework. This substitution results in a matrix that is highly resistant to heavy metal leaching [18,19,21].
5. Coordination Bonds/Chemical Complexation:
  Some metal ions (e.g., Cu^2+^ and Zn^2+^) can form coordination bonds with functional groups present in the geopolymer structure [18,19,20,22]. Specifically, they interact with the oxygen atoms of the non-condensed silanol (-Si-OH) and aluminol (-Al-OH) groups, resulting in a stable metal–hydroxyl complex.

In the work of Jiang et al., the bauxite-tailing geopolymer immobilizes Pb^2+^ and Cu^2+^ mainly by forming Si–O–M and Al–O–M covalent bonds that integrate the metals into the aluminosilicate network, and by physically encapsulating them within a dense, amorphous gel [23]. In the highly alkaline synthesis environment, Pb^2+^ also precipitates as Pb(OH)_2_ and Cu^2+^ can form CuO, which becomes trapped in the matrix [23].

Even in the work of Zhang et al., the rare-earth-tailing geopolymer captures Pb^2+^ and Cd^2+^ through similar Si–O–M/Al–O–M bonds (T–O–M) and by electrostatic attraction, where the divalent cations replace Na^+^ at negatively charged [AlO_4_]^−^ sites [24]. Both systems show a homogeneous distribution of the metals in the gel, confirmed by XPS and elemental mapping, indicating that the metals are incorporated rather than forming separate crystalline phases [24]. Consequently, immobilization efficiencies exceed 96% for Pb^2+^ and Cu^2+^ in the bauxite system and >99% for Pb^2+^ and 92–96% for Cd^2+^ in the rare-earth system, even under acidic leaching conditions [23,24].

These mechanisms chemically bind the contaminants to prevent their release into the environment, offering superior immobilization due to the molecular structure of the geopolymers.

The dissolution process promotes the conversion of octahedral [Al(VI) and pentahedral [Al(V)] species into tetrahedral [Al(IV)] units, which are charge-balanced by alkali cations. This [Al(IV)] dominated structure forms the backbone of the aluminosilicate framework, providing reactive binding sites for heavy metals and improving immobilization efficiency [22].

To establish the mechanism correlation, recent X-ray absorption spectroscopy (XAS) studies have provided direct evidence of metal incorporation within this framework. Specifically, analysis of metakaolin-based geopolymers containing Cr(III) confirmed that chromium exists solely in the trivalent state after geopolymerization [22]. Extended X-ray absorption fine structure (EXAFS) analysis further revealed that the Cr(III) site is octahedrally coordinated by six oxygen atoms in the first shell (CrO_6_). Crucially, the EXAFS data demonstrated a strong non-direct Cr–Al interaction in the second coordination shell. This structural feature is attributed to the formation of Cr–O–Al bridging structures (atomic triplets with an oxygen bridge). This spectroscopic observation definitively shows that the units are chemically trapped within the aluminosilicate network, often resulting in the formation of a zeolitic cage structure [22]. This strong chemical bonding confirms the direct link between the polycondensation of the framework and the creation of highly favorable sites for the chemical entrapment and long-term stabilization of metal species.

A novel approach to enhance metal ion retention involves the use of chemical agents: chelating agents like sodium diethyl dithio carbamate (C_5_H_10_NS_2_Na, DTC) introduce -S and -N donor atoms or electrons forming stable complexes with cations (e.g., Ni^2+^, Cd^2+^, Cr^3+^), lowering their mobility [25], its macromolecular nature leads to a direct physical influence on the evolving geopolymer structure improving compressive strength due to macromolecules physically adsorption by C–S–H gels, and intercalate into the interlayer of the C-S-H gels, which changes the structure of these gels as shown in the work of Guo et al. (2017) [25].

Reducing agents such as sodium sulfide (Na_2_S) convert redox-sensitive species, transforming mobile and toxic Cr(VI) anions into the less soluble Cr(III) cations, which then precipitate or become structurally incorporated into the geopolymer network [26,27,28]. Redox reactions involving sulfide (S^2−^) lead to the generation of significant amounts of hydroxyl ions (OH^-^), as shown in the reaction equations [26,29]:

3S^2−^ + 8CrO_4_^2−^ + 20H_2_O → 3SO_4_^2−^ + 8Cr^3+^ + 40OH^−^

3S^2−^ + 4Cr_2_O7^2−^ + 16H_2_O → 3SO_4_^2−^ + 8Cr^3+^ + 32OH^−^

The ions produced in these reduction reactions act to further promote the synthesis of the metakaolin-based geopolymer. This promotion occurs in the early stages of the geopolymerization process (dissolution) by increasing the alkalinity necessary for precursor dissolution [26,29]. Adsorbents like hydroxyapatite (Ca_5_(PO_4_)_3_(OH), Hap) in geopolymers stabilize heavy metals through cation exchange, where its Ca^2+^ exchange with metals like Cu^2+^ [30]. According to Billah et al., at a pH greater than 9, typical of alkali-activated materials, Hap becomes deprotonated [31], which can be advantageous for the adsorption of cations such as Ni^2+^.

These coupled processes lead to near-complete immobilization of Ni, Cd and Zn from incineration fly ash and up to 99% retention of Cr(VI) [25,26]. However, such synergistic methods have so far been applied mainly to geopolymers where heavy metals were introduced as salts, such as Na_2_CrO_4_ or PbCrO_4_ [29], or to wastes like chromite ore processing residue (COPR), which contains substantially lower Cr concentrations compared with GSW (5.72 wt% vs. about 20 wt% on average in GSW) [26]. A significant research gap remains in systematically applying combined geopolymerization and chemical stabilization to industrial sludges characterized by complex composition and extremely high heavy metal loads, especially when processed as received to minimize pre-treatment energy costs. This synergistic treatment relies on solid-state chemical reactions that are difficult to assess stoichiometrically within complex, heterogeneous waste matrices. Consequently, a critical factor requiring optimization is the effective ratio between the chemical agent and the specific industrial waste, which is essential to achieve robust and cost-effective immobilization performance [25].

This study investigates metakaolin-based geopolymers for the stabilization/solidification (S/S) of an industrial galvanic sludge (referred to as DE), incorporating separately chelating (DTC), reducing (Na_2_S), and adsorbent (Hap) agents. Industrial waste was used as received to reduce energy consumption in the event of scaling up the process. The amounts of reactive agents (DTC, Na_2_S and Hap) were chosen to be in a large molar ratio with Cr and Ni cations and were kept at the same mass ratio with DE to allow a quick replacement in the event of a scaled-up process. The selected molar ratios of the chemical agents were intentionally set above the theoretical stoichiometric requirements for two reasons. First, the industrial sludge has compositional heterogeneity, which is typical for such complex waste streams. The use of an excess of chemical agents ensures sufficient dosage to effectively target the heavy metals of interest (Cr and Ni) throughout the entire, non-uniform matrix. Second, the geopolymeric environment contains various dissolved ionic species that could potentially participate in competitive reactions with the chemical agents, potentially reducing their treatment efficiency in Cr and Ni retention. Although a molar ratio higher than the stoichiometric requirement was selected for the reduction of Cr(VI) through Na_2_S addition, the S^2−^/Cr(VI) molar ratio was maintained at 3.5. This ensures an excess of reducing agent to fully convert Cr(VI), while remaining below the molar ratio of 6 reported by Guo et al. (2017) to avoid the formation of crystalline phases such as Na_2_SO_4_, which can compromise the structural stability of the material [25]. The effectiveness of these geopolymers in limiting Cr and Ni leaching is evaluated, offering a sustainable approach for the management of this type of hazardous waste.

## 2. Materials and Methods

In this study, we used ARGICAL™ M1000 metakaolin (MK) (Imerys, Paris, France). According to the manufacturer’s data, the chemical composition of MK is 55 wt% SiO_2_, 40 wt% Al_2_O_3_, 1.4 wt% Fe_2_O_3_, 1.5 wt% TiO_2_, 0.8 wt% Na_2_O + K_2_O, 0.3 wt% CaO + MgO, and 1 wt% loss on ignition at 1000 °C. After drying, DE lost ~78 wt% due to moisture, and its total metal content was assessed on the dry DE by X-Ray fluorescence (Zetium XRF, Malvern Panalytical, Malvern, Worcestershire, UK) by the “Omniam” method. Dried DE contains high concentrations of Cr_2_O_3_ (40.01 wt%), NiO (18.06 wt%), and Fe_2_O_3_ (4.68 wt%). The complete chemical characterization is reported in Table S1 of the Supplementary Materials. Alkaline activator was prepared by dissolving laboratory-grade NaOH pellets (96%, Sigma–Aldrich, Milan, Italy) in a commercial sodium silicate solution (Na–Silicate) (Ingessil Srl, Verona, Italy; SiO_2_/Na_2_O = 3.0, 26.50 wt% SiO_2_, 8.70 wt% Na_2_O; density 1.368 g/cm^3^and pH = 11.7 at 20 °C). Hydrated sodium sulfide (Na_2_S, min. 30% water, ITW Reagents, Milan, Italy), sodium dithiocarbamate (DTC; (C_2_H_5_)_2_NCS_2_Na·3H_2_O, Carlo Erba, Milan, Italy), and hydroxyapatite (Hap, Ca_5_(OH)(PO_4_)_3_, Sigma-Aldrich, Milan, Italy) were used separately as chemical agents. The geopolymer synthesis followed an established procedure [32,33]: pre-prepared alkaline activator (NaOH + Na–Silicate aged 24 h) was mixed with metakaolin for 5 min. The chemical agent was first mixed with the as-received DE sludge in distilled water and stirred for 1 h. This mixture was then incorporated into the geopolymer paste and mixed for a further 5 min. After degassing on a vibrating table, the paste was cast, sealed-cured (48 h), demoulded, and then ambient-cured until analysis after 28 days. Samples were cured at room temperature at 100% relative humidity. The dosage of DE was maintained at 5 wt% in all formulations. This dosage was selected based on findings from our previous work [32], which indicated that higher additions of this waste (10 wt%) significantly compromise the structural integrity and mechanical stability of the resulting geopolymer matrix. Since the primary objective of this study is to isolate and evaluate the specific efficacy of chemical agents in enhancing the retention of Cr and Ni, a constant, low waste loading was essential. This approach ensures that any improvement in metal retention can be attributed primarily to the chemical stabilization mechanisms, rather than to variations in the physical encapsulation efficiency of the geopolymer, which is highly dependent on the waste content. The composition of the various formulations is provided in Table 1. Fourier-transform infrared (FT-IR) (Prestige21 Shimadzu spectrophotometer, Shimadzu Italia s.r.l., Milan, Italy, equipped with a deuterated triglycine sulfate detector and KBr windows) spectra were recorded to assess geopolymerization (from 4000 to 370 cm^−1^ at a resolution of 2 cm^−1^ over 60 scans). A leaching test was carried out according to UNI EN 12457–2:2004 [34] using ICP–MS iCAPTQ (Thermo Fisher Scientific, Waltham, MA, USA). Compressive strength was measured on cubic specimens (Instron 5567 Universal Testing Machine, Norwood, MA, USA). Tests were performed at a crosshead speed of 1 mm/min with a maximum applied load of 30 kN. Microstructural analysis was performed using a scanning electron microscope with Field Emission Gun SEMFEG (Nova NanoSEM 450, Bruker Corp., Billerica, MA, USA), equipped with energy dispersive spectroscopy (EDS). Data analysis was performed using Origin 9.1 software. Bulk density (ρ_b_), was measured using Micrometrics Geopyc 1360 (Micrometrics Instruments, Norcross, GA, USA). True density (ρ_t_), was measured through a helium pycnometer (Micrometrics Accupyc 1330, Micrometrics Instruments, Norcross, GA, USA) using a weighted amount of a pulverized sample.

## 3. Results

### 3.1. Assessing the Impact of DE and Chemical Agents on Geopolymerization

**Figure 1:**
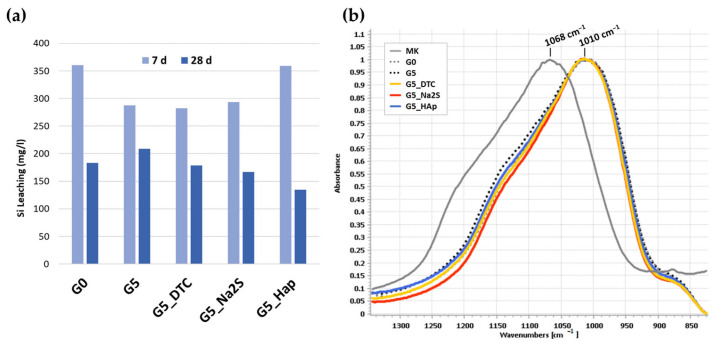
(**a**) Silicon leaching release after 7 and 28 days of curing; (**b**) FT-IR spectra in the 1500–800 cm^−1^ region showing Si–O–T band shift.

The impact of DE and chemical agents on geopolymerization was evaluated through silicon leaching tests, FT-IR spectroscopy, compressive strength measurements and microstructural analysis (Figure 1). In Figure 1a, the Si leaching values decreased in all samples from 7 to 28 days of curing, indicating progressive geopolymerization and an increase in the cross-linking degree of the Si–O–T network (T=Si or Al^−^(IV)) [16]. After 28 days, Si release values were comparable across all formulations (ranging from 140 to 200 ppm), suggesting that neither the addition of 5 wt% DE nor the chemical agents significantly hindered the geopolymerization [35]. Different chromium species influence the polymerization degree and structural stability of the Si-O bonds [25]. Cr^3+^ cations are readily attracted and immobilized by the electronegative aluminum–oxygen unit ([-OAl(OH)_3_]^−^) of the geopolymer structure during polymerization. However, high concentrations of Cr^3+^ can destabilize the network by forming long weak Cr-[AlO_4_] bonds when participating in charge balance for alumina tetrahedra, leading to a dramatic decrease in compressive strength and slowing down the reaction process. Conversely, less soluble chromium forms, Cr(VI) are mainly stabilized through physical encapsulation [25].

Nickel (Ni^2+^) is typically stabilized through physical encapsulation, precipitation (as hydroxides due to the alkaline environment), and ion exchange with alkali cations within the [AlO_4_]^−^ network [18]. The immobilization also involves coordination with silanol (-Si-OH) and aluminol (-Al-OH) groups present in the geopolymer gel. When heavy metals like are incorporated via complex waste, such as galvanic sludge at low dosage (5 wt%), the resultant reduction in mechanical strength (−24.3%) is primarily attributed to structural inhomogeneity caused by the introduction of unreacted waste particles of DE waste, rather than chemical interference that inhibits the dissolution or polymerization of the Si-Al phases. Therefore, the sustained low release confirms that the bulk geopolymerization reaction proceeds efficiently, providing the robust Si-O-Al backbone necessary for the successful immobilization of both and species.

This result is corroborated by the FT-IR analysis (Figure 1b). The characteristic Si–O–T band of metakaolin, initially centered at 1068 cm^−1^ before alkali activation, shifted to approximately 1010 cm^−1^ in all geopolymer samples [16,36,37]. This shift confirms the successful incorporation of Al in its IV-fold coordination into the silicate network [38]. No significant spectral differences were observed between the reference sample and those containing DE or chemical agents, indicating that the low dosage (5 wt%) of DE did not alter the reaction mechanism. However, higher DE additions (10–20%), as reported in our previous study [32], resulted in a smaller shift (to 1016 and 1021 cm^−1^, respectively) and a substantial reduction in the compressive strength (by 38.6% and 81.9%) with respect to the sample without DE addition, in this case, the smaller shift in the Si-O-T band compared to the geopolymer without DE addition (G0) indicates that the incorporation of Al (IV) into the geopolymer matrix is somewhat hindered by the excessive addition of DE (10–20%), contrary to what occurs with a low DE addition (5%), confirming that excessive waste content decrease geopolymerization degree [39]. Complete FT-IR spectra of all the samples investigated are provided in Figure S1.

It was found that geopolymerization is not hindered by the incorporation of 5 wt% DE or by the addition of chemical agents, which allows for the isolated assessment of the efficacy of chemical additives in enhancing the immobilization of heavy metals within the DE-containing geopolymer.

**Figure 2:**
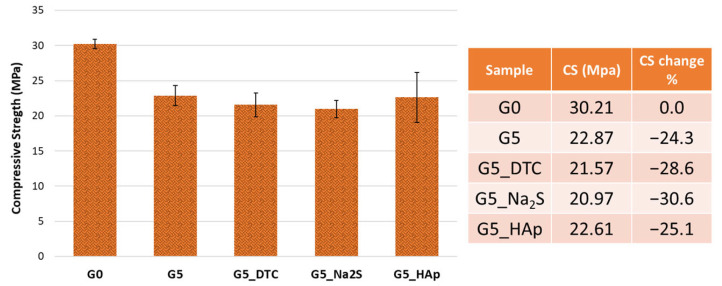
Compressive strength of the geopolymer samples.

Although the compressive strength of samples containing 5 wt% DE and chemical agents decreased by 24–30% compared to the waste-free reference (G0) (Figure 2), this reduction is likely not due to inhibited geopolymerization, as said before. On the contrary, it may be attributed to the introduction of unreacted DE particles, which increase structural inhomogeneity, or to the high-water content (78% moisture) of DE, which increases the overall water-to-solid ratio and negatively affects mechanical properties [16] leading to higher total porosity ash shown in Table 2. Nevertheless, all materials exhibited good mechanical performance, with compressive strength values exceeding 20 MPa, consistent with conventional geopolymers used in S/S applications [40]. The presence of DTC leads to lower porosity, but not to an increase in compressive strength; probably its macromolecular nature fills the pores by a physical interaction, intercalating into the interlayer of the gels as observed by Guo et al. (2017) [25].

**Figure 3:**
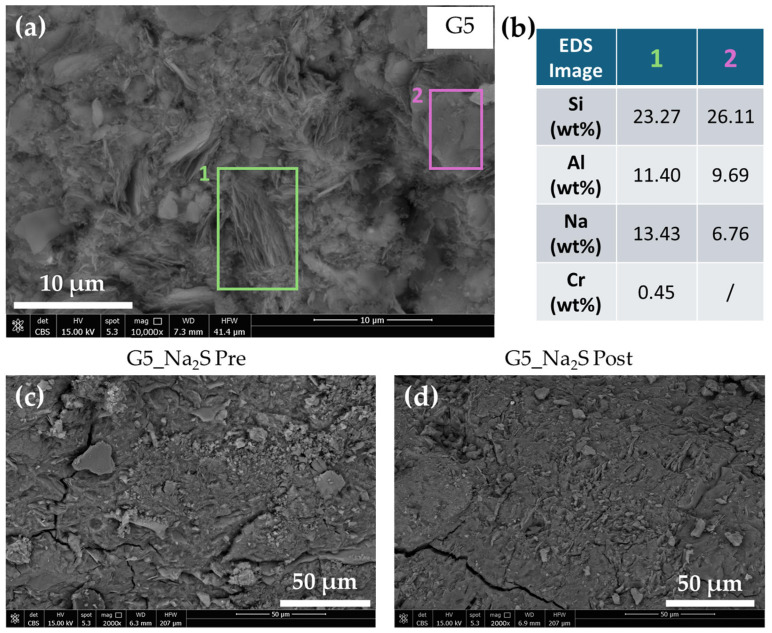
SEMFEG micrographs (BSE detector) of the geopolymer samples: (**a**) G5 at 10,000× magnification; (**b**) G5 EDS analysis; (**c**) G5_Na_2_S before leaching test; (**d**) G5_Na_2_S after leaching test.

Figure 3a shows the SEM micrograph (BSE detector) of the G5 sample, taken at 10,000× magnification. A needle-like structure can be observed, which EDS analysis (Figure 3b) confirms to be the geopolymeric gel incorporating the DE waste, as already observed by the authors in Cr-tannery wastewater containing geopolymers [41]. This is evidenced by the chemical analysis, which reveals a chromium content of 0.45 wt%, consistent with the theoretical Cr content of the G5 geopolymer (approximately 5000 ppm). The same micrograph also shows the composition of an area of the geopolymeric gel without acicular crystals. In this region, no chromium was detected, and the Si, Al, and Na values, the main constituents of the geopolymer matrix, align with those calculated in Table 1. Given that the G5 formulation inherently contains chromium predominantly as the highly mobile, anionic species Cr(VI) (chromate, CrO_4_^2−^), the observed localization must be explained by the challenging nature of Cr(VI) immobilization in geopolymers.

The fundamental geopolymer structure, composed of aluminosilicate units, carries an overall negative charge, particularly on the aluminum tetrahedra [AlO_4_]^-^. Consequently, Cr(VI) anions are subject to electrostatic repulsion from the geopolymer gel. This inherent repulsion explains the result of no Cr being detected in the homogeneous, non-acicular bulk gel areas.

Figure 3c,d show the SEMFEG micrographs of the G5_Na_2_S sample, taken before and after the leaching test, respectively. These images were obtained to assess the structural integrity of the geopolymer containing both the waste and the reducing agent when immersed in water. As can be seen in the two micrographs, there are no significant morphological differences before and after leaching in water, which confirms the good structural stability of the matrix despite the addition of DE waste and Na_2_S.

### 3.2. Role of Chelating, Reducing, and Adsorbent Agents on Heavy Metal Leaching DE-Geopolymers

**Figure 4:**
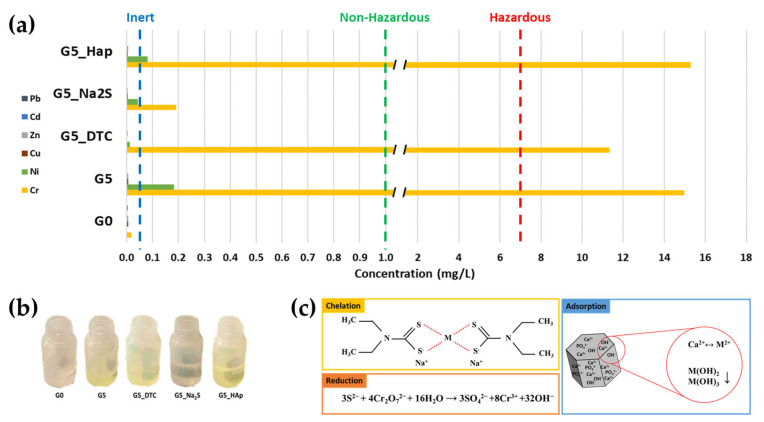
(**a**) Leaching test results (UNI EN 12457-2:2004 [34]) of heavy metals from geopolymers compared to Directive (EU) 2018/850 limits; (**b**) Visual appearance of leachates from geopolymer samples after leaching tests; (**c**) Interaction mechanism of the three chemical agents with heavy metals.

Incorporation of DE, rich in Cr (26.22 wt%) and Ni (13.26 wt%), into geopolymers G5 led to leaching concentrations of Cr 14.972 ppm and Ni 0.182 ppm, as shown in Figure 4a (leaching test values of the samples are reported in Table S2 of the Supplementary Materials), classifying the material as hazardous waste according to Directive (EU) 2018/850. Although the overall retention percentages were satisfactory, above 99% for Ni and 89% for Cr, calculated from their concentrations in the different samples, further reduction in leaching was required to enable the material’s disposal as non-hazardous waste. The lower retention of Cr (89% in reference sample G5) compared to Ni within the geopolymer matrix can be attributed to its chemical speciation. In the DE waste, chromium is present in its hexavalent form, Cr(VI). Under the highly alkaline conditions typical of geopolymer synthesis, Cr(VI) exists as oxyanions (chromate, CrO_4_^2−^; dichromate, Cr_2_O_7_^2−^). The negatively charged aluminosilicate network of the geopolymer is known to be less effective in immobilizing anions due to electrostatic repulsion [21]. Consequently, the primary mechanism for Cr(VI) retention in this system is not chemical binding but rather physical encapsulation within the geopolymeric matrix.

Separate addition of chemical agents (DTC, Na_2_S and Hap) improved the immobilization efficiency. DTC in G5_DTC substantially decreased Ni release from 0.182 to 0.012 ppm corresponding to a 93.40% reduction in leaching as shown in Table 3, owing to the strong complexation of Ni^2+^, which readily forms square-planar complexes [42]. Ni(II)(DTC)_2_ is a highly stable complex, with an overall formation constant of log β_2_ ≈ 21.5 [43]. β_2_ indicates the stability constant, which refers to the formation of the 1:2 complex, where one Ni^2+^ ion coordinates two DTC^−^ ligands, which expresses the thermodynamic stability of the complex:

$$\beta_{2}=\frac{\left[Ni{\left(DTC\right)}_{2}\right]}{\left[{Ni}^{2+}\right]{\left[{DTC}^{-}\right]}^{2}}$$

In aqueous media at alkaline pH, Ni(II)(DTC)_2_ is sparingly soluble and readily precipitates, which explains its effective immobilization within the geopolymer matrix (pH > 11) [43]. DTC only slightly reduced Cr leaching (14.972 to 11.338 ppm). Conversely, Na_2_S (G5_Na_2_S) drastically decreased Cr release from 14.972 ppm to 0.191 ppm, corresponding to a 98.72% reduction in Cr leaching, consistent with its ability to reduce Cr(VI) to Cr(III) [26]. This was corroborated by leachate color changes (yellow to colorless, shown in Figure 4b), as the presence of chromate/dichromate anions (CrO_4_^2−^, Cr_2_O_7_^2−^) was eliminated [44], favoring encapsulation of Cr(III) cations within the geopolymer network. Finally, Hap (G5_Hap) improved Ni immobilization (0.182 to 0.081 ppm), which corresponds to a 55.49% decrease in leaching, likely through cation exchange with Ca^2+^ in its lattice and precipitation as hydroxide [45], but worsened Cr leaching from 14.972 ppm to 15.283 ppm. According to Billah et al. [31], the adsorption of Cr(VI)O_4_^2−^ onto Hap is highly pH-dependent. The process is most efficient at acidic pH values between 3 and 6, where the Hap surface is protonated, generating positively charged sites that attract CrO_4_^2−^ anions. At higher pH values, pH > 9, typical of geopolymer matrices, the surface of Hap becomes deprotonated and negatively charged. This results in electrostatic repulsion with Cr(VI)O_4_^2−^ present in DE, and consequently, a marked decrease in adsorption efficiency. The different mechanisms involved are schematically summarized in Figure 4c.

Considering the retention performance of the different stabilizing agents shown in Table 3, it is evident that under the extremely alkaline pH conditions typical of a geopolymer and with this DE waste containing Cr(VI) and Ni, the best approach is to use a reducing agent such as Na_2_S for Cr(VI) and DTC for Ni. However, for these agents, it must be assessed whether their stability remains unaltered in the long term. For Hap, the conditions described are not ideal for ensuring efficiency in retaining these heavy metals, primarily due to the highly alkaline environment.

## 4. Conclusions

This study successfully demonstrates a low-temperature, low-carbon strategy for the stabilization/solidification of galvanic sludge waste using metakaolin-based geopolymers combined with chemical agents. The direct use of 5 wt% as-received sludge, without thermal pre-treatment, aligns with practical industrial needs. The geopolymeric matrix maintained its structural integrity, achieving compressive strengths > 20 MPa, while effectively immobilizing critical pollutants.

The incorporation of chemical agents drastically reduced leaching: Na_2_S achieved a −98.7% reduction in Cr leaching by reducing Cr(VI) to the less soluble Cr(III), and DTC enabled a −93.4% reduction in Ni leaching through stable complex formation. In contrast, Hap was only effective for Ni.

Na_2_S is highly toxic and corrosive, posing risks to the environment because it releases hydrogen sulfide (H_2_S), which is extremely toxic. Na_2_S is highly soluble and can increase sulfide concentrations in water. From the cost point of view, it is inexpensive compared to many alternative reducing agents and largely available, but if it is industrially used, the costs for handling and storage are high because it needs corrosion-resistant equipment and H_2_S detection and ventilation. From an industrial perspective, the preferred source of sulfide will therefore be S^2−^ supplied by blast-furnace slag rather than pure Na_2_S. In practice, sulfide is introduced via slag, which releases S^2−^ gradually during geopolymerisation [29], providing the needed reductive capacity while avoiding the safety and environmental issues associated with Na_2_S, and future development should focus on integrating slag-derived sulfide into the binder matrix. In the present study, Na_2_S was employed solely as a laboratory probe to verify whether the reduction mechanism works on a real waste stream, namely galvanic sludge. Demonstrating successful Cr(VI) reduction with Na_2_S establishes the feasibility of the chemistry before transitioning to the more sustainable, slag-based S^2−^ approach envisioned for future industrial implementations.

In the case of the total lifecycle cost, it can be high due to environmental control tools, waste management, and regulatory compliance. For all these reasons, the research will continue by using greener reducing agents such as FeSO_4_.

DTC is a technically effective chelating agent for Nickel. Therefore, for the treatment of dangerous industrial waste, as the wastes studied in this paper, it can be useful if used in closed, well-controlled industrial systems, where waste streams can be safely managed.

In future work, the behavior of the matrix under different extraction scenarios can also be explored. The synergistic combination of geopolymerization and chemical stabilization presents a highly efficient and sustainable alternative to conventional high-temperature treatments, offering a promising pathway for the safe management of electroplating wastes.

## Figures and Tables

**Table 1:** Geopolymer formulations and labeling. Oxides molar ratios were kept constant in all the samples (Na_2_O/Al_2_O_3_ = 0.82, SiO_2_/Al_2_O_3_ = 3.46, SiO_2_/Na_2_O = 4.21).

Sample Name	MK [g]	NaOH Pellets [g]	Na-Silicate [g]	DE [g]	H_2_O [g]	DTC [g]	Na_2_S [g]	Hap [g]
G0	70	9.8	70	/	14	/	/	/
G5	70	9.8	70	3.5	14	/	/	/
G5_DTC	70	9.8	70	3.5	14	1.05	/	/
G5_Na_2_S	70	9.8	70	3.5	14	/	1.05	/
G5_Hap	70	9.8	70	3.5	14	/	/	1.05

**Table 2:** Bulk density (ρ_b_), true density (ρ_t_), and total porosity values were calculated on the basis of the density values according to the formula: (1 − ρ_b_/ρ_t_)·100.

Sample Name	Bulk Density (ρ_b_) (g/cm^3^)	True Density (ρ_t_) (g/cm^3^)	Total Porosity (%)
G0	1.3963 ± 0.0024	2.0412 ± 0.0015	31.59
G5	1.4063 ± 0.0026	2.0870 ± 0.0009	32.62
G5_DTC	1.4180 ± 0.0030	1.9972 ± 0.0021	29.00
G5_Na_2_S	1.4133 ± 0.0048	2.0861 ± 0.0009	32.25
G5_Hap	1.4040 ± 0.0019	2.0606 ± 0.0018	31.86

**Table 3:** Variation % in leaching values for samples containing stabilizing agents relative to the G5 reference.

Sample	Cr (%)	Ni (%)
G5	0	0
G5_DTC	−24.3	−93.5
G5_Na2S	−98.7	−75.9
G5_Hap	+2.1	−55.3

## Data Availability

The original contributions presented in this study are included in the article/Supplementary Material. Further inquiries can be directed to the corresponding authors.

## Supplementary Materials

The following supporting information can be downloaded at: https://www.mdpi.com/article/10.3390/polym18010028/s1, Table S1. XRF chemical composition of dried DE (moisture content ~78 wt%). Table S2. Leaching test results (EN 12457 [34]) of heavy metals from geopolymers compared to Directive (EU) 2018/850 limits. Figure S1. Complete FT-IR spectra of the investigated samples in the range 4000–370 cm^−1^

## References

[1] Pérez-Villarejo L., Martínez-Martínez S., Carrasco-Hurtado B., Eliche-Quesada D., Ureña-Nieto C., Sánchez-Soto P.J. (2015). Valorization and Inertization of Galvanic Sludge Waste in Clay Bricks. Appl. Clay Sci..

[2] Silva J.E., Paiva A.P., Soares D., Labrincha A., Castro F. (2005). Solvent Extraction Applied to the Recovery of Heavy Metals from Galvanic Sludge. J. Hazard. Mater..

[3] Silva J., Soares D., Paiva A., Labrincha J., Castro F. (2005). Leaching Behaviour of a Galvanic Sludge in Sulphuric Acid and Ammoniacal Media. J. Hazard. Mater..

[4] Magalhães J. (2004). Effect of Experimental Variables on the Inertization of Galvanic Sludges in Clay-Based Ceramics. J. Hazard. Mater..

[5] Rossini G., Bernardes A. (2006). Galvanic Sludge Metals Recovery by Pyrometallurgical and Hydrometallurgical Treatment. J. Hazard. Mater..

[6] Hills C.D., Pollard S.J.T. (1997). The Influence of Interference Effects on the Mechanical, Microstructural and Fixation Characteristics of Cement-Solidified Hazardous Waste Forms. J. Hazard. Mater..

[7] Luz C., Rocha J., Cheriaf M., Pera J. (2006). Use of Sulfoaluminate Cement and Bottom Ash in the Solidification/Stabilization of Galvanic Sludge. J. Hazard. Mater..

[8] Garcia-Valles M., Avila G., Martinez S., Terradas R., Nogués J.M. (2007). Heavy Metal-Rich Wastes Sequester in Mineral Phases through a Glass–Ceramic Process. Chemosphere.

[9] Turner L.K., Collins F.G. (2013). Carbon Dioxide Equivalent (CO_2_-e) Emissions: A Comparison between Geopolymer and OPC Cement Concrete. Constr. Build. Mater..

[10] Duxson P., Mallicoat S.W., Lukey G.C., Kriven W.M., Van Deventer J.S.J. (2007). The Effect of Alkali and Si/Al Ratio on the Development of Mechanical Properties of Metakaolin-Based Geopolymers. Colloids Surf. A Physicochem. Eng. Asp..

[11] Duxson P., Fernández-Jiménez A., Provis J.L., Lukey G.C., Palomo A., Van Deventer J.S.J. (2007). Geopolymer Technology: The Current State of the Art. J. Mater. Sci.

[12] Nath P., Sarker P.K. (2014). Effect of GGBFS on Setting, Workability and Early Strength Properties of Fly Ash Geopolymer Concrete Cured in Ambient Condition. Constr. Build. Mater..

[13] Amran Y.H.M., Alyousef R., Alabduljabbar H., El-Zeadani M. (2020). Clean Production and Properties of Geopolymer Concrete; A Review. J. Clean. Prod..

[14] McLellan B.C., Williams R.P., Lay J., Van Riessen A., Corder G.D. (2011). Costs and Carbon Emissions for Geopolymer Pastes in Comparison to Ordinary Portland Cement. J. Clean. Prod..

[15] Siva Shankari S.V., Sivasakthi M. (2025). A State-of-the-Art Review on Effectiveness of Geopolymer Technology Toward Dye Degradation, Heavy Metal Encapsulation and Its Future Prospects on Environmental Remediation. Environ. Qual. Manag..

[16] Khale D., Chaudhary R. (2007). Mechanism of Geopolymerization and Factors Influencing Its Development: A Review. J. Mater. Sci..

[17] Tian Q., Bai Y., Pan Y., Chen C., Yao S., Sasaki K., Zhang H. (2022). Application of Geopolymer in Stabilization/Solidification of Hazardous Pollutants: A Review. Molecules.

[18] Genua F., Lancellotti I., Leonelli C. (2025). Geopolymer-Based Stabilization of Heavy Metals, the Role of Chemical Agents in Encapsulation and Adsorption: Review. Polymers.

[19] Vu T.H., Gowripalan N. (2018). Mechanisms of Heavy Metal Immobilisation Using Geopolymerisation Techniques—A Review. ACT.

[20] El-Eswed B.I., Aldagag O.M., Khalili F.I. (2017). Efficiency and Mechanism of Stabilization/Solidification of Pb(II), Cd(II), Cu(II), Th(IV) and U(VI) in Metakaolin Based Geopolymers. Appl. Clay Sci..

[21] Provis J.L. (2009). Immobilisation of Toxic Wastes in Geopolymers. Geopolymers.

[22] Giorgetti M., Berrettoni M., Aquilanti G., Boldrini G., Lancellotti I., Leonelli C. (2020). The Coordination Core and Charge of Chromium in Metakaolin-Geopolymers as Revealed by X-Ray Absorption Spectroscopy. Mater. Lett..

[23] Jiang J., Luo H., Wang S., Ou X., Su J., Lyu Z., Chen J., Wei D. (2024). Synthesis of Tailing Slurry-Based Geopolymers for the Highly Efficient Immobilization of Heavy Metals: Behavior and Mechanism. Appl. Clay Sci..

[24] Zhang B., Yu T., Deng L., Li Y., Guo H., Zhou J., Li L., Peng Y. (2022). Ion-Adsorption Type Rare Earth Tailings for Preparation of Alkali-Based Geopolymer with Capacity for Heavy Metals Immobilization. Cem. Concr. Compos..

[25] Guo X., Zhang L., Huang J., Shi H. (2017). Detoxification and Solidification of Heavy Metal of Chromium Using Fly Ash-Based Geopolymer with Chemical Agents. Constr. Build. Mater..

[26] Sun T., Chen J., Lei X., Zhou C. (2014). Detoxification and Immobilization of Chromite Ore Processing Residue with Metakaolin-Based Geopolymer. J. Environ. Chem. Eng..

[27] Chen J., Wang Y., Wang H., Zhou S., Wu H., Lei X. (2016). Detoxification/Immobilization of Hexavalent Chromium Using Metakaolin-Based Geopolymer Coupled with Ferrous Chloride. J. Environ. Chem. Eng..

[28] Chen J., Wang Y., Zhou S., Lei X. (2017). Reduction/Immobilization Processes of Hexavalent Chromium Using Metakaolin-Based Geopolymer. J. Environ. Chem. Eng..

[29] Zhang J., Provis J.L., Feng D., Van Deventer J.S.J. (2008). The Role of Sulfide in the Immobilization of Cr(VI) in Fly Ash Geopolymers. Cem. Concr. Res..

[30] Arokiasamy P., Abdullah M.M.A.B., Arifi E., Razak R.A., Rojviriya C., Mydin M.A.O., Sandu A.V., Yaacob N.A., Mohamed R. (2025). Hydroxyapatite Incorporated Geopolymer Porous Adsorbent for Efficient Removal of Copper Ions and Ciprofloxacin. J. Am. Ceram. Soc..

[31] Billah R.E.K., Khan M.A., Park Y.-K., Am A., Majdoubi H., Haddaji Y., Jeon B.-H. (2021). A Comparative Study on Hexavalent Chromium Adsorption onto Chitosan and Chitosan-Based Composites. Polymers.

[32] Genua F., Giovini M., Santoni E., Berrettoni M., Lancellotti I., Leonelli C. (2025). Factors Affecting Consolidation in Geopolymers for Stabilization of Galvanic Sludge. Materials.

[33] Russo R.E., Santoni E., Fattobene M., Giovini M., Genua F., Leonelli C., Lancellotti I., Herrero A., Berrettoni M. (2025). Design of Experiments Approach for Efficient Heavy Metals Stabilization Using Metakaolin-Based Geopolymers. Molecules.

[34] (2004). Characterisation of Waste. Leaching. Compliance Test for Leaching of Granular Waste Materials and Sludges.

[35] Lancellotti I., Kamseu E., Michelazzi M., Barbieri L., Corradi A., Leonelli C. (2010). Chemical Stability of Geopolymers Containing Municipal Solid Waste Incinerator Fly Ash. Waste Manag..

[36] Ryu G.S., Lee Y.B., Koh K.T., Chung Y.S. (2013). The Mechanical Properties of Fly Ash-Based Geopolymer Concrete with Alkaline Activators. Constr. Build. Mater..

[37] Lee W.K.W., Van Deventer J.S.J. (2003). Use of Infrared Spectroscopy to Study Geopolymerization of Heterogeneous Amorphous Aluminosilicates. Langmuir.

[38] Wan Q., Rao F., Song S., García R.E., Estrella R.M., Patiño C.L., Zhang Y. (2017). Geopolymerization Reaction, Microstructure and Simulation of Metakaolin-Based Geopolymers at Extended Si/Al Ratios. Cem. Concr. Compos..

[39] Wang T., Rao F., Yang L., Jiang K., Lin N., Mo L. (2024). Influence of Wastes and Synthesis Conditions on the Compressive Strength, Setting Time and Gels of Waste-Based Geopolymers. Gels.

[40] Fan C., Wang B., Ai H., Qi Y., Liu Z. (2021). A Comparative Study on Solidification/Stabilization Characteristics of Coal Fly Ash-Based Geopolymer and Portland Cement on Heavy Metals in MSWI Fly Ash. J. Clean. Prod..

[41] Boldrini G., Sgarlata C., Lancellotti I., Barbieri L., Giorgetti M., Ciabocco M., Zamponi S., Berrettoni M., Leonelli C. (2021). Efficient chemical stabilization of tannery wastewater pollutants in a single step process: Geopolymerization. Sustain. Environ. Res..

[42] Khan M.d.N.I., Fackler Jnr J.P., Murray H.H., Heinrich D.D., Campana C. (1987). Structure of the Beta Form of Bis(Diethyldithiocarbamato)Nickel(II). Acta Crystallogr. C Cryst. Struct. Commun..

[43] Labuda J., Skatuloková M., Németh M., Gergely S. (1984). Formation and Stability of Diethyldithiocarbamate Complexes. Chem. Zvesti.

[44] Tumolo M., Ancona V., De Paola D., Losacco D., Campanale C., Massarelli C., Uricchio V.F. (2020). Chromium Pollution in European Water, Sources, Health Risk, and Remediation Strategies: An Overview. Int. J. Environ. Res. Public Health.

[45] Ferri M., Campisi S., Gervasini A. (2019). Nickel and Cobalt Adsorption on Hydroxyapatite: A Study for the de-Metalation of Electronic Industrial Wastewaters. Adsorption.

