# Supplementary material for: Chelating, Reducing, and Adsorbing Agents in Geopolymers for Heavy Metals Stabilization from Galvanic Sludge
Source: Polymers (Basel). 2025 Dec 22;18(1):28. doi: 10.3390/polym18010028 (PMC12788012; doi:10.3390/polym18010028)
Supplement: Supplementary file 1 [file polymers-18-00028-s001.zip › polymers-3990613-supplementary.pdf]

Supplementary Material

Table S1. XRF chemical composition of dried DE (moisture content ~78 wt%).

Component	wt%	Component	wt%
**Cr_2O_3**	40.01	**CuO**	0.18
**NiO**	18.06	**P_2O_5**	0.15
**Fe_2O_3**	4.68	**Zn**	583 ppm
**SO_3**	3.63	**Sn**	445 ppm
**Na_2O**	2.74	**Al**	278 ppm
**Cl**	2.02	**Pb**	143 ppm
**CaO**	1.77	**Sr**	67 ppm
**MgO**	0.99	**LOI[a]**	25.18
**SiO_2**	0.45		

[a]Loss of Ignition at 1000°C.

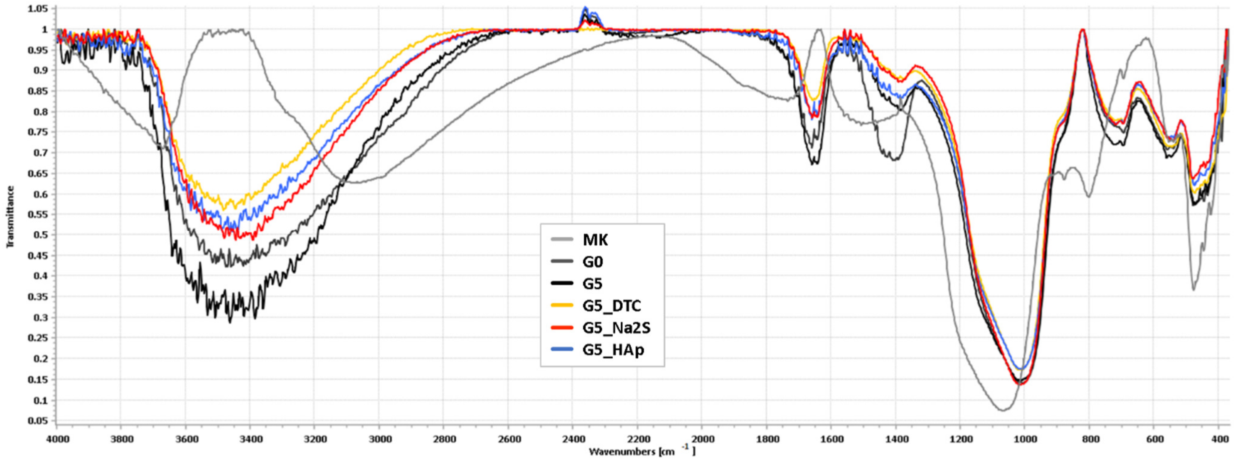

Figure S1. Complete FT-IR spectra of the investigated samples in the range 4000–370 cm^{-1}.

Table S2. Leaching test results (EN 12457) of heavy metals from geopolymers compared to Directive (EU) 2018/850 limits.

Samples Metals	G0 C_L (mg/L)	G5 C_L (mg/L)	G5_DTC C_L (mg/L)	G5_Na_2S C_L (mg/L)	G5_HAp C_L (mg/L)	Inhert	Not dangerous	Dangerous
Cd	<LOD	<LOD	<LOD	<LOD	<LOD	0.004	0.1	0.5
Cr	0.019	14.972	11.338	0.191	15.283	0.05	1	7
Cu	0.006	0.007	0.001	0.005	0.005	0.2	5	10
Ni	<LOD	0.182	0.012	0.044	0.081	0.04	1	4
Pb	0.003	0.002	<LOD	0.002	0.001	0.05	1	5
Zn	0.005	0.006	0.004	0.005	1.03	0.4	5	20
